# Role of ciliary dysfunction in a new model of obesity and non-alcoholic steatohepatitis: the *foz/foz* mice

**DOI:** 10.1186/2049-3258-72-S1-O7

**Published:** 2014-06-06

**Authors:** Laurence Poekes, Vanessa Legry, Geoffrey Farrell, Isabelle Leclercq

**Affiliations:** 1 Laboratory of Hepato-Gastroenterology, UCL, Brussels, Belgium; 2 ANU, Canberra, Australia

## Introduction

*Foz/foz* mice are deficient for Alms1, a ubiquitous protein essential for proper primary cilium function. They are prone to insulin resistance, obesity and diabetes, a phenotype accelerated by high-fat diet (HFD) feeding. Their unique metabolic phenotype has been linked to hyperphagia resulting from abnormal ciliary function in the central nervous system [1]. The aim of our study is to verify the dependence of the phenotype on over-feeding and to explore the role of Alms1 deficiency in intestinal energy absorption.

## Materials and methods

Male *foz/foz* (Alms1-/-) and wild-type (WT) littermates were fed a HFD for 4 weeks to evaluate their food intake, metabolic parameters (glucose tolerance, steatosis, adiposity) and tissue inflammation. We next performed a pair-feeding experiment in which *foz/foz* mice had access to the exact same amount of HFD consumed by WT the day before. Lipid absorption was evaluated by oral fat tolerance test and total lipid content in the feces.

## Results

As expected, *foz/foz* mice ate more (18.2 vs 14.1 kcal/d, p<0.001), became more obese (42.5 vs 26.8g, p=0.01) and glucose intolerant than WT mice fed a HFD (p=0.008). Unlike WT mice, they also developed steatosis, adipose tissue and liver inflammation. In the pair-feeding experiment, *foz/foz* mice and WT mice were fed iso-calorically. However, *foz/foz* mice gained more weight (+54.3% vs +29.7%, p<0.001), were more glucose intolerant and presented higher adipose inflammation than WT mice. To explain the metabolic alterations, we hypothesized that Alms1 deficiency in intestine could contribute to increased nutrient absorption. We found that fecal lipid content was lower in *foz/foz* than in WT mice matched for HFD intake (31.3 vs 45.9 mg/24h feces, p=0.01). Moreover, upon oral fat load, *foz/foz* mice had higher plasma triglyceride levels than WT mice (79.1 vs 15.7 mg/dL, p< 0.05).

## Conclusion

These results suggest that, beside causing hyperphagia, Alms1 deficiency increased dietary energy extraction, that could participate to the metabolic phenotype leading to insulin resistance and obesity. The understanding of the mechanisms at play may uncover new potential therapeutic targets.
